# Diseases of the Skin

**Published:** 1899-01

**Authors:** 


DISEASES OF THE SKIN. An Outline of the Principles and Practice of Dermatology. By MALCOLM MORRIS, F. R. C. S., Surgeon to the Skin Department, St. Mary's Hospital, London; Corresponding Member of the K. K. Gesellschaft Der Aerzte in Wien, of the Wiener Dermatologische Gesellschaft, and of the Société Française dê Dermatologie. With 10 colored plates and 26 engravings. New and revised edition. Philadelphia: Lea Brothers & Co. 1898.

The first edition of Malcolm Morris's work appeared in 1894, in the form of a volume of 556 pages, illustrated with eight chromolithographs and seventeen wood-cuts devoted to the clinical facts, and the principles of treatment of skin diseases, based on the results of modern research as to their causes. The book now before us, similarly entitled, has attained the size of 589 pages, and contains ten colored plates and twenty-six engravings, thoroughly, revised, partly rewritten, added to, and enhanced by giving the treatment after the description of the several diseases, instead of being included in one chapter as was the case in the former edition. The work is essentially clinical and practical in its scope and is characterised throughout for its clearness and simplicity of style, direction of diction, and strong common sense.

As a guide to furnishing useful information in the science of dermatology, it is alike suitable for the student, physician and specialist. E. W.

